# Accessible art in healthcare facilities: exploring perspectives of healthcare art for visually impaired people

**DOI:** 10.3389/fmedt.2023.1205361

**Published:** 2023-10-23

**Authors:** Daryia Palityka, Evangelia Chrysikou, Niamh Murtagh

**Affiliations:** The Bartlett School of Sustainable Construction, University College London, London, United Kingdom

**Keywords:** healthcare facility, hospital, art, accessibility, visual impairment, museum

## Abstract

**Introduction:**

Art in healthcare facilities shows promising results in improving patients' health and well-being and, as such, meets the WHO's definition of health technology. Yet, it remains unclear if healthcare art equally benefits all users. Given the growing number of visually impaired people (VIP), it is valuable to determine whether healthcare art is accessible to VIP and to explore strategies for improving it.

**Methods:**

This study employed a mixed methodology, which included (1) secondary research of 25 cases of healthcare art programmes to identify the presence of accessible art in healthcare facilities and the practices that influence it; (2) review of thirty-one Health Building Notes and four supplementary British guidelines on healthcare art to discover if the accessibility of art is required and identify which recommendations influence it; and (3) interview surveys of healthcare art practitioners from three London NHS Trusts to identify opportunities to increase arts accessibility.

**Results and discussion:**

The evidence showed that healthcare art programmes were mostly inaccessible to VIP. Most healthcare art programmes did not involve VIP in the commissioning process and, thus, lacked procedures that could facilitate accessibility. There were not enough recommendations in the healthcare facility guidelines to support the accessibility of arts for VIP. The recommendations on artwork in healthcare facility guidelines could increase accessibility if particular conditions were met. Interviews with NHS trusts in London revealed numerous opportunities to improve arts accessibility for healthcare art programmes.

## Introduction

Healthcare facilities accommodate the largest proportion of people with different types of disabilities. To support patients' health and well-being, medical architecture should go hand in hand with human physiological and psychological needs (1–3). Accessibility of hospitals is one of the primary approaches to adapting environments for different needs and providing equality of care (4). The UK National Health Service (NHS) is committed to providing accessible facilities for its users and meeting the Equality Act 2010 (5). Different solutions within healthcare facilities and their services were proposed to improve people with disabilities' experiences of healthcare services. Architecture and design can be altered by including wheelchair access, tactile signage, colour contrast in finishes, as well as adequate acoustics and lighting (6, 7). Services can be more easily accessible through numerous types of information channels, including comprehensive orientation for access and use of facilities (8, 9). However, there is still much room for improvement: NHS Property services reported that the largest proportion of patients with disabilities (78%) had difficulties in accessing their facilities and only a third of NHS trusts fully complied with the Accessible Information Standard (10–12). Overall, the research literature on the accessibility of healthcare facilities is very scarce, which requires studies of different design elements (4).

In the past 30 years, it has been proven that the clinical environment has a significant influence in facilitating treatment processes. Art integrated into healthcare spaces is one of the components that enhances hospital users' experiences. Art is an essential component of the therapeutic clinical environment that can serve as a landmark to assist patients with wayfinding, as a positive distraction in examination and treatment rooms or waiting areas, and as an element that helps to integrate or instead escape the hospital walls (13–16). Two prominent organisations, Paintings in Hospitals (London) and Hospital Art (Manchester), now known as Arts for Health, were founded in 1959 and 1973 respectively (17, 18). Since that time, healthcare art developed into numerous art programmes in different NHS trusts that included not only receptive but also participatory arts. Currently, there is a vast body of evidence that healthcare art is not only beneficial for itself, but generates therapeutic effects in conjunction with the physical environment. In fact, art has been shown to provide various benefits in the healthcare built environment, including decreasing stress and anxiety levels among both patients and staff, enhancing the quality of sleep, reducing the need for pain and sleep medication, shortening hospital stays, and promoting better communication between patients and staff (14, 19–22). Not only are these benefits valuable for the well-being of healthcare facility users, but they can also be advantageous for the healthcare system by reducing the hospital maintenance costs (23).

Since healthcare art is primarily a visual aesthetic modality, it is yet unknown if it is accessible for various user groups, such as persons with visual disabilities. As the population ages, the prevalence of visual impairment will inevitably increase. By 2050, it's estimated nearly one in five UK citizens will have some degree of vision loss (24, 25). Furthermore, the prevalence of eye diseases will likely be greatly influenced by the high rates of obesity and diabetes (26–30). It is also crucial to note that multimorbidity is more common in persons with disabilities than it is in people without eyesight problems, they are more likely to require mental health help, and the majority of sight loss occurs in dementia patients (31–34). In the context of healthcare in the UK, the prevalence of VIP suggests that approximately 1 in every 40 registered patients may have significant sight problems, highlighting the importance of accessibility and tailored support in different types of medical settings (35). Therefore, it is critical to ensure that healthcare facilities of different types are accessible since VIP may require medical assistance for both their visual impairment and other medical conditions they may have.

Older adults represent the largest group of people with visual impairments in the UK, and comprise the majority of visitors to galleries and museums (36–38). This required adaptations of services to the needs of older VIP. “Accessible art” offers a solution of experiencing art for people with disabilities by adapting fine arts through enhancing visual stimuli for those who can partly see and adding either audio, tactile, smell, or, sometimes, taste triggers to deliver art messages (39–41). Some of the latest reviews on evidence-based healthcare art, discusses effects of art on human preferences, mental and physical state (14, 19). However, no research has been performed on accessible arts in healthcare facilities. Therefore, it is needed to study if art in hospitals suits the needs of individuals with visual disabilities to ensure equal access to the benefits of healthcare arts and support the protected characteristics outlined in the Equality Act 2010.

Given that visual art is an integral part of a favourable healthcare environment and the rising numbers of people with VIP within the health service, this research aim is to identify if the accessibility of art for individuals with visual disabilities is considered in healthcare environments and what opportunities exist to increase it. This paper will present an overview of accessible art solutions that exist in museums and galleries, forming a basis for healthcare art programmes analysis. Based on that knowledge, healthcare art programmes and healthcare facilities guidelines will be evaluated to see if accessibility of art is considered in healthcare. Additionally, we will perform case studies of healthcare art programmes with interviews of senior professionals working on art to identify opportunities for healthcare art programmes in terms of their accessibility. The study is crucial as it will reveal the current situation regarding the accessibility of the arts in healthcare institutions, laying the groundwork for further study in this area. The study will also help healthcare art practitioners to get insights into accessible solutions for their programmes and support compliance with the Equality Act. The condition of healthcare arts and their impact on those with visual impairments may, subsequently, be favourably influenced.

### Literature review of accessible arts in museums and galleries

Although healthcare built environments serve a population with a higher prevalence of disabilities, the conversation around accessible art in these settings has been relatively limited. As art continues to exhibit its potential to promote healing and enhance well-being in healthcare settings, ensuring accessibility for individuals with diverse needs becomes imperative. While the discourse on accessible art in museums and galleries has led to the development of innovative and inclusive strategies for displaying and interpreting art for people with disabilities. This highlights the potential for knowledge transfer and exchange of ideas between the art and healthcare fields, with lessons learned from museums and galleries informing the design and implementation of accessible art programmes in healthcare settings.

### Tactile arts

The perceptual needs of VIP are well studied and considered in transforming visual art forms to adapt to VIP's sensory needs. One of the most common solutions for making art accessible to VIP is haptic or tactile art. Tactile art can either represent a translation of a visual artwork or be an original art piece made to be touched. The translations may include tactile representations of visual pictures; exact or small copies of original sculptures; replicas or three-dimensional models of objects depicted in a painting (42–44). The creation process of tactile art requires a sophisticated blend of materials and processes that are thoughtfully tailored to enhance VIP sensory experience. Artists often utilise a range of materials, such as various types of textured papers, fabrics, and sculpting mediums and even use of 3D-printed textures (45–47). Techniques like embossing, relief carving, and layering, and 3D-printing are commonly employed to provide depth and complexity to the surface of the artwork (48). This approach not only allows VIP to engage with the art through touch but also stimulates their imagination, enabling them to create a mental image of the artwork in a manner that is uniquely personal. Moreover, not only are tactile arts beneficial on their own as aesthetic objects, but they also provide a new means of communication between sighted and blind people (49). As a result, tactile art can provide access for blind people to visual arts and make the arts more inclusive, giving the opportunity for people of different abilities to enjoy art and interact via tactile representations.

### Touching concerns

Although tactile art is one of the well-developed accessible art solutions for VIP, COVID-19 pandemics needed new solutions to continue touching experiences for VIP. Several museums and galleries adapted their services to new realities. For instance, they implemented reusable tactile handouts featuring raised-line images of original visual artworks. This approach allowed individuals personal access to tactile art experiences while facilitating subsequent disinfection upon return, circumventing the need for widespread use of a single tactile representation, and enabling the maintenance of social distancing (50). Furthermore, the Henry Moore Gallery in London devised an engaging solution with their “This Living Hand” exhibition. Here, visitors were encouraged to interact with sculptures after cleansing their hands using an original washbasin installed at the gallery entrance. Apart from the tactile arts on display, the washbasin itself was an artwork, crafted from a single piece of rock (51). Finally, the options proposed far more from current pandemics to protect art pieces and support visitors' hygiene included using nitrile gloves, hand wipes, and taking rings off before interacting with a tactile art piece (52, 53). Thus, despite difficulties arising in recent years, some solutions might help continue tactile experiences for VIP.

### Hearing

Another method that helps VIP perceive art is the use of sound. One of the well-developed adaptations is audio descriptions of art pieces that can be either delivered by an exhibitor or listened to as a recording from different digital devices. The scope of the available options is extensive: from simple verbal descriptions of static art to artists’ explanations of an art piece, historical notes, and ambient sounds in the background (54, 55). This approach has been demonstrated to be efficacious in providing visually impaired individuals with auditory descriptions of visual art and enabling them to experience and appreciate artwork through their imagination. Furthermore, the utilisation of assistive technology, such as handheld devices or mobile applications, allows visually impaired individuals to engage with art autonomously, thereby promoting positive effects on their well-being through a sense of control (56, 57). Furthermore, adding sound effects to visual artwork descriptions is greatly appreciated by people with normal vision, conveying other sides of the emotional essence of a work of art (58).

### Other senses and multisensory art

Given the connectivity of the human brain and the intimate relationship between all the senses, a multisensory experience is the one that is most valued among accessible arts (59). The aforementioned approach has become more popular in recent years with technological development (58, 60–62). One of the most popular combinations is tactile objects, such as tactile pictures, samples of art, replicas of objects depicted in the artwork, and audio descriptions (63). An alternative form of representation involves he utilisation of three-dimensional images with sensors that evoke verbal descriptions or sounds pertaining to particular parts of an artwork (64). More sophisticated combinations might involve touch, smell, hearing and even taste by combining different materials and techniques and sometimes converting the whole space for multisensory installation (65–67). Multisensory arts and exhibits are greatly appreciated by VIP providing them with aesthetic experiences and a sense of independence, which is extremely valuable for them (68).

### VIP engagement in the creation of accessible art

Moreover, VIP engagement in the arts commissioning process can ensure the appropriateness of art and the formulation of best practices for art inclusivity. Anne Chick identified principles of co-production with VIP as the main stakeholders that included co-creation and co-assessment meetings for establishing multisensory exhibits (68). The essential principles for fostering inclusive and effective participatory experiences included meticulous planning, material and space accessibility, and the iterative nature of co-creation. In these meetings, the artists generated ideas together with VIP, where the main methods to improve communication were the transcription of texts into large text and audio formats and the creation of small-scale artefacts to identify ideas for further improvements. The result determined working approaches that helped art professionals deliver a VIP-centred art exhibition, followed by co-assessment sessions to identify ideas for further improvements. Moreover, the co-production principles helped to give voice to VIP, and respond to their needs, build a dialogue with the community, and develop new skills in participants. Interestingly, such workshop sessions might be not only valuable for the successful results but also therapeutic for VIP as participatory art sessions that could create a sense of belonging and stimulate self-expression and socialisation (69).

### Guidance for museums and galleries

A significant role in art accessibility projects in the UK is played by governmental and charitable organisations, such as the Arts Council, the Royal National Institute of the Blind (RNIB), and VocalEyes (53, 70, 71). In addition to providing funding and support, these organisations offer recommendations to improve accessibility, such as the Talking Images Guide by VocalEyes, which provides practical suggestions for planning accessible exhibits, creating inclusive events, and enhancing the physical environment (53). Another example of accessible art recommendations was published by RNIB in “Shifting Perspectives” report based on planning for accessibility in the London 2012 Cultural Olympiad. It focused on such activities as consultation with VIP, providing audio-description, tactile exhibits, and large print information, training museum staff and volunteers, including case studies of museums and galleries that have implemented accessibility measures successfully, offering practical examples and inspiration for other institutions (72). Thus, recommendations and guidance offered by organisations such as the Arts Council, RNIB, and VocalEyes are essential for improving accessibility and inclusivity in the arts and culture sector for VIP.

Accessible art programmes have emerged as effective mechanisms for promoting inclusivity and engagement in diverse settings. In museum and gallery contexts, initiatives such as the “Touch Tours” programme at the Metropolitan Museum of Art in New York and the “Touch Tours for All!” at the Tate Modern in London showcase the potential of tactile art to provide VIP with tangible connections to renowned artworks (73, 74). In recent years, there has been a notable surge in the popularity of immersive exhibitions that engage many senses since they have been recognised for their capacity to democratise art and foster more visitor involvement (58). These exhibitions are particularly inclusive and enriching for both the general population and VIP. In healthcare settings, the integration of multisensory art, as seen in such facilities as St. James's Hospital in Ireland, Guy's and St. Thomas’ Hospital, and Moorfields Eye Hospital in the UK and Duke University Hospital's in the US, offers therapeutic benefits for patients in various spaces (75–77). By engaging different senses, these initiatives alleviate stress and anxiety, fostering a healing environment that extends beyond traditional medical practices. Both contexts demonstrate the wider community impact of accessible art, fostering awareness and empathy for diverse sensory experiences while contributing to more inclusive environments. Through these successful implementations, accessible art programmes have proven to be transformative tools that bridge gaps, inspire creativity, and nurture well-being in various sectors.

Overall, this overview of accessible arts in museums and galleries provides insights on accessible art forms for VIP, some issues to consider and the actors that can help to support the creation of inclusive art projects. Two main themes will support the research: (1) accessible art forms that will be used to compare with arts in healthcare schemes and evaluate the degree of accessibility, and (2) stakeholders involved in the arts commissioning to evaluate activities that help to achieve accessibility of arts. Moreover, insights gained from guidance on creating accessible art exhibitions in museums and galleries will serve as a foundation for identifying potential solutions for healthcare art accessibility for individuals who are visually impaired or blind.

## Research methodology

**Figure 1:**
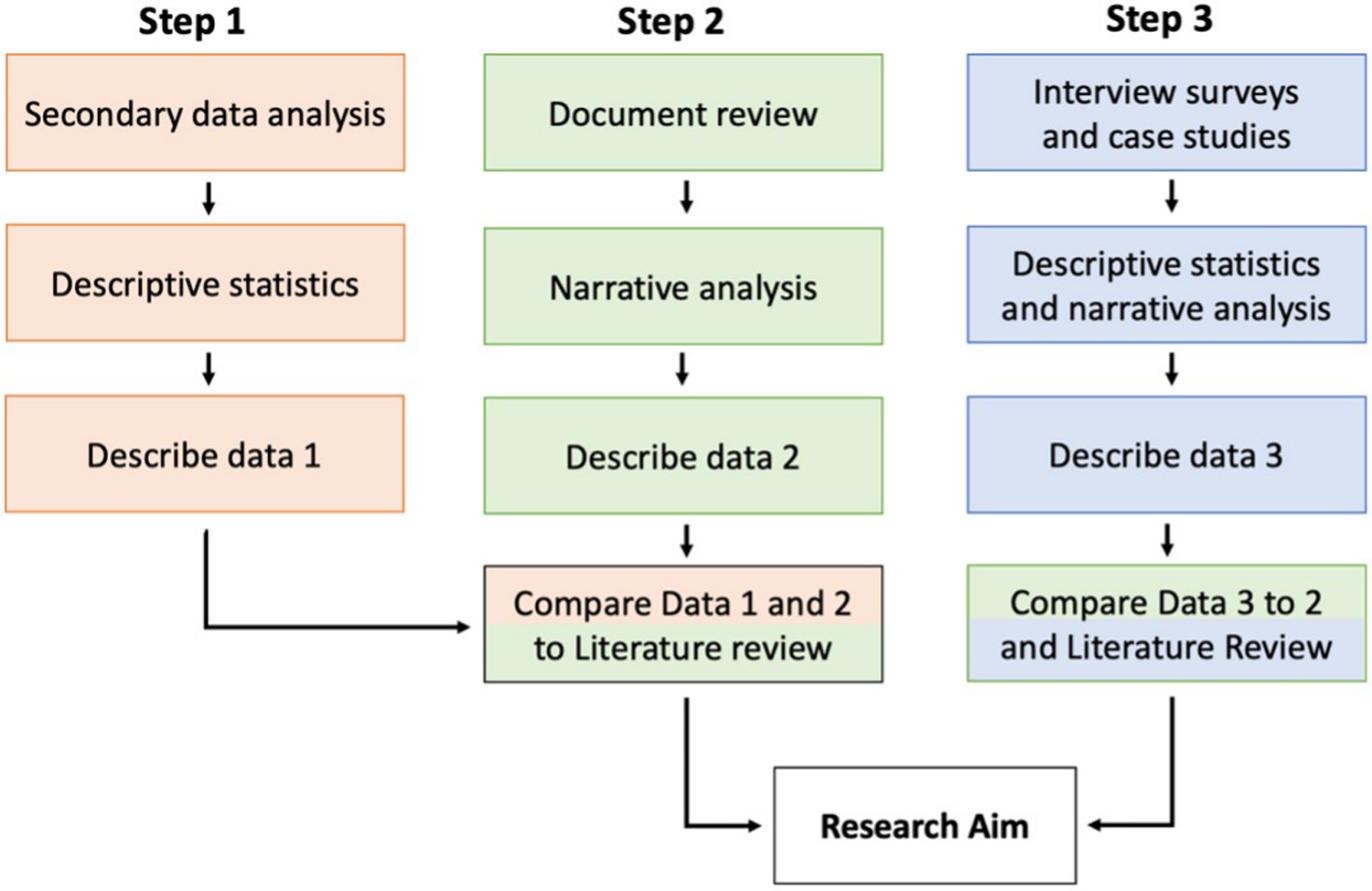
Research steps with data collection, analysis, and results.

The study was conceived as a qualitative exploratory research initiative to investigate whether accessible art for visually impaired persons (VIP) is considered in healthcare environments and explore opportunities to enhance its accessibility. The research strategy selected for this study is a mixed method research including secondary research of healthcare art programmes, review of healthcare facilities guidelines, and case studies of healthcare art programmes (Figure 1). A mixed-method strategy was selected to explore the field of accessible art for VIP from a comprehensive perspective, as no previous studies have been conducted on this topic and establish a foundation for future research.

### Secondary research of art programmes in healthcare facilities

The first research method (Step 1) involved a qualitative secondary analysis of art programmes. The purpose of the first step was to determine whether accessible art for VIP exists in healthcare facilities, as well as to identify the practices that facilitate or hinder its implementation. The secondary analysis was identified to be the optimal approach for gathering enough data from healthcare facilities in the most cost- and time- effective manner.

The secondary data on healthcare art programmes has been collected through Google Search as the primary search engine using the following search keywords: “art in healthcare facilities/hospitals”, “NHS healthcare art programmes”, “accessible art in healthcare facilities/hospitals”. Secondary data contained documents and websites with textual, visual, and video information about art schemes with sufficient information to evaluate the overall state of the art program. The inclusion criteria were all art projects in British healthcare facilities.

**Figure 2:**
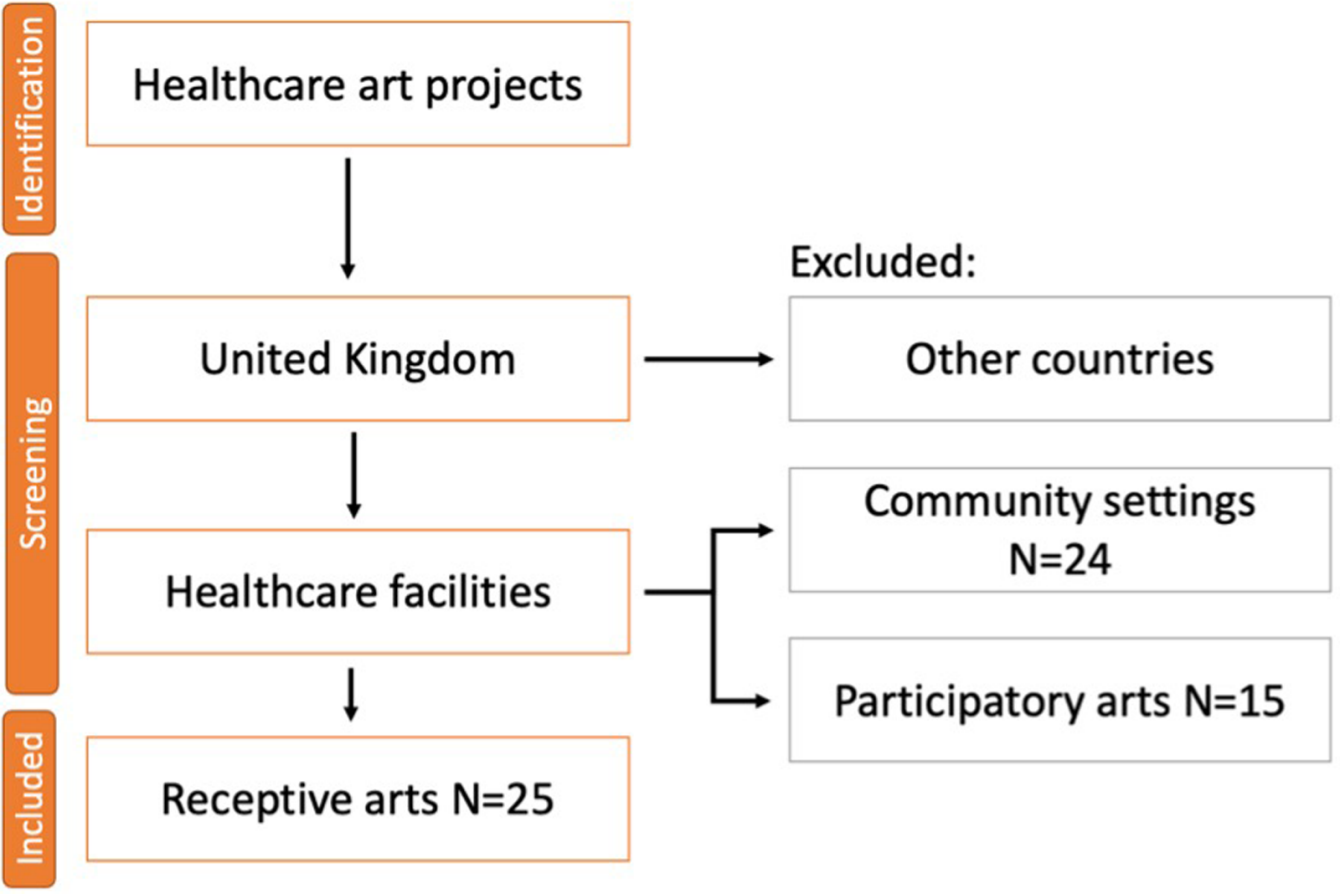
Flow diagram of secondary data inclusion process.

Secondary data inclusion process is presented in the Figure 2. After initial Internet search, a number of papers and websites were excluded from the study as they were not based in the UK. In sum, there were found three major healthcare art reports with case studies of art programmes across the country that formed the basis for investigation. Five additional websites of NHS trusts and art charities completed the secondary data sample. The next step was filtering data in terms of the type of the arts they provided: receptive or participatory. As a result, fifteen participatory art projects were excluded from the study and 25 projects on receptive art in healthcare facilities were included in further evaluation.

To categorize the collected data, the framework of the literature review on accessible art in museums and galleries was utilized, which consisted of two main themes for ensuring the accessibility of art for VIP: (1) the availability of accessible art forms (such as tactile, audio, and multisensory) to assess the level of accessibility, and (2) the involvement of stakeholders in the arts co-creation process to evaluate activities that could facilitate or hinder the attainment of accessibility in arts.

### Review of healthcare facilities guidelines for the art accessibility requirements

The second research strategy (Step 2) involved a review of healthcare estates guideline to explore the peculiarities of art commissioning and maintenance in healthcare settings, and identify any restrictions, considering the specifics of healthcare space, that might prevent the implementation of accessible art for VIP. Additionally, this step aimed to assess regulators' viewpoints on the need for the art accessibility for VIP in healthcare facilities. A document review method was selected as it allowed to research the broader perspective on arts accessibility for different types of facilities across the country. Health Building Notes (HBN) were chosen as the primary source for guidance evaluation, given their provision of best practice guidelines on healthcare facility design and planning for all NHS Trusts, and the fact that art is an integral part of the healthcare built environment (15). In addition, it is noteworthy that HBN are commonly regarded by NHS trusts as non-mandatory guidelines, albeit with some degree of informality. This, in turn, has a bearing on the decision-making process regarding healthcare estate projects and budgetary allocation, with potential implications for the accessibility of healthcare arts.

HBN were accessed through official NHS website. The current NHS collection included 31 HBN guidance documents, with some supplemental materials for the main documents. Following the examination of the HBN guidelines, four supplementary references were identified that were repeatedly cited in HBN as sources of good practice in healthcare arts. These references were analysed in the same manner as HBN. First, the reviewed documents were evaluated regarding the general content on arts and built environment considerations for art commissioning process. The requirements were grouped into several categories, i.e., dimensions, based on their similarities in content, which identified major focus points in the guidelines. The dimensions were then analysed to determine how they might influence the accessibility of arts. Second, the documents were analysed regarding the presence of any considerations on art accessibility for VIP. Two themes identified in the literature review were used for accessibility evaluation: the requirement for accessible art forms and the need for stakeholders' engagement in the art commissioning process. The following search keywords were used in each document: “art/s accessibility/inclusivity”, “art/s for sensory disabled/impaired/blind/visually impaired/visually disabled/low sighted”; “users/patients/staff engagement/collaboration”.

### Interview surveys with healthcare art professionals

The final step of the research (Step 3) entailed conducting **online interview surveys** with healthcare art programme professionals to identify opportunities to increase the art accessibility for VIP. Given the exploratory nature of the research, the interview surveys provided an opportunity to examine a wide range of perspectives on accessible solutions for art. The use of interview surveys facilitated the standardisation of responses from NHS professionals from different types of healthcare facilities. By employing a structured questionnaire, all participants were posed the same questions in the same manner, thereby reducing bias, and enhancing the reliability of the data. Furthermore, the utilization of interview surveys emerged as the most optimal approach in terms of time and resources, given the COVID-19 pandemic's constraints and ethical considerations. Additionally, informal discussions with interviewees provided insights into the proposed solutions and informed data analysis, leading to further discussion.

**The survey design** was informed by the information derived from two guidance documents on accessible art for VIP from the literature review section: “*Museums, Galleries and Heritage Sites: Improving access for blind and partially sighted people. The Talking Images Guide*” (53); and “*Shifting Perspectives. Opening up Museums and Galleries to Blind and Partially Sighted People*” (72). Drawing on these resources ensured that the survey questions were grounded in established best practices for improving art access for VIPs. Specifically, the survey questions were designed to explore which accessible art opportunities derived from the guidance for accessible arts in museums and galleries could be applied in healthcare facilities. This approach allowed the research to examine the feasibility and appropriateness of existing recommendations in healthcare settings. Additionally, the survey sought to identify any solutions that were already in practice in healthcare facilities, thus providing a more nuanced understanding of the current state of accessible art in healthcare. The survey delineated four primary steps to guide the development of an accessible art program: (1) planning for inclusion, (2) improving access through information, (3) improving access through art services and forms, and (4) welcoming visitors. The activities constituting these steps are presented in the subsequent section. The interviewed professionals were asked to reply whether an offered activity is either already introduced in their art program, is possible/impossible to introduce, or is uncertain. The complete survey questionnaire can be accessed in the Appendix.

Participant selection for the interviews was based on purposive sampling to ensure that only those with expertise and experience in accessible healthcare art projects were included in the study. This approach facilitated the gathering of insights about the integration of accessible solutions in healthcare settings. The sample included senior healthcare professionals such as managers and directors from healthcare facilities that had implemented at least one accessible art project identified in Step 1 of the study. Ethics approval was obtained for data collection, and all interviews were conducted anonymously. A total of three professionals agreed to participate in the study and were administered interview surveys, followed by informal discussions via Zoom. To supplement the survey data with original examples of accessible art projects, case studies of selected trusts were conducted and presented alongside the interview survey results.

### Data analysis

In step 1 of the study, the secondary data were analysed through a content analysis method (78, 79). The initial coding process involved the categorization of the materials found. The initial codes underwent refinement in accordance with the findings of the literature review, which encompassed best practices pertaining to the accessibility of the arts within museum and gallery settings. The data was ultimately encoded according to the predetermined categories, which included the form of art (such as sculpture, painting, digital art, etc.), the sensory modalities affected (including visual, audio, tactile, multisensory, etc.), and the level of community engagement (whether it involved the general population, VIP, or neither). A descriptive analysis was conducted to summarise the coded data and determine the prevalence of various forms of art and their associated accessibility activities.

In Step 2 of the study, a narrative approach was employed to analyse data extracted from the Health Building Notes (HBN) and four supplementary documents focused on healthcare arts (80, 81). Initial analysis involved examining the general content pertaining to art requirements within healthcare facilities, aiming to identify the main themes in these requirements. Subsequently, a content analysis was conducted to ascertain the presence of accessibility requirements for VIP. The primary coding scheme encompassed several categories developed based on the literature review and the above-mentioned review of the documents, including accessibility requirements, types of art, sensory modalities, implementation guidelines, and community engagement. However, upon conducting the analysis, it was discovered that the documents lacked specific details regarding some of the codes. As a result, the coding system underwent refinement to specifically target accessibility requirements and community engagement. The coded data were summarised to identify the prevalence of requirements concerning arts accessibility for VIP.

In Step 3, a mixed-methods approach was employed to analyse interview surveys. Descriptive statistics were used to quantitatively assess the prevalence of accessible art activities among the selected trusts. Further insights into accessible solutions for healthcare institutions were provided by the analysis of case studies on accessible art projects using a narrative method.

## Results

### Analysis of healthcare art projects in British healthcare facilities

Secondary data research was performed to discover whether the accessible art for VIP takes place in healthcare facilities in the UK and whether stakeholders are included in the art commissioning process.

The included secondary data were evaluated using two themes identified in the literature review: (1) accessible for VIP art forms and (2) stakeholder engagement in the arts commissioning. For the first theme, arts were considered accessible for VIP if they integrated at least one additional sense along with the sense of sight: touch, sound, smell, or taste. For example, this could be tactile elements in a visual art piece, interactive art, or sound installations. For the second theme, data on stakeholders in art programmes were primarily available about users' engagement. As a result, “VIP engagement” was evaluated as a second criterion to examine strategies influencing art's accessibility. Apart from those two main criteria, the type of healthcare facility that provided the art programme was recorded.

**Figure 3:**
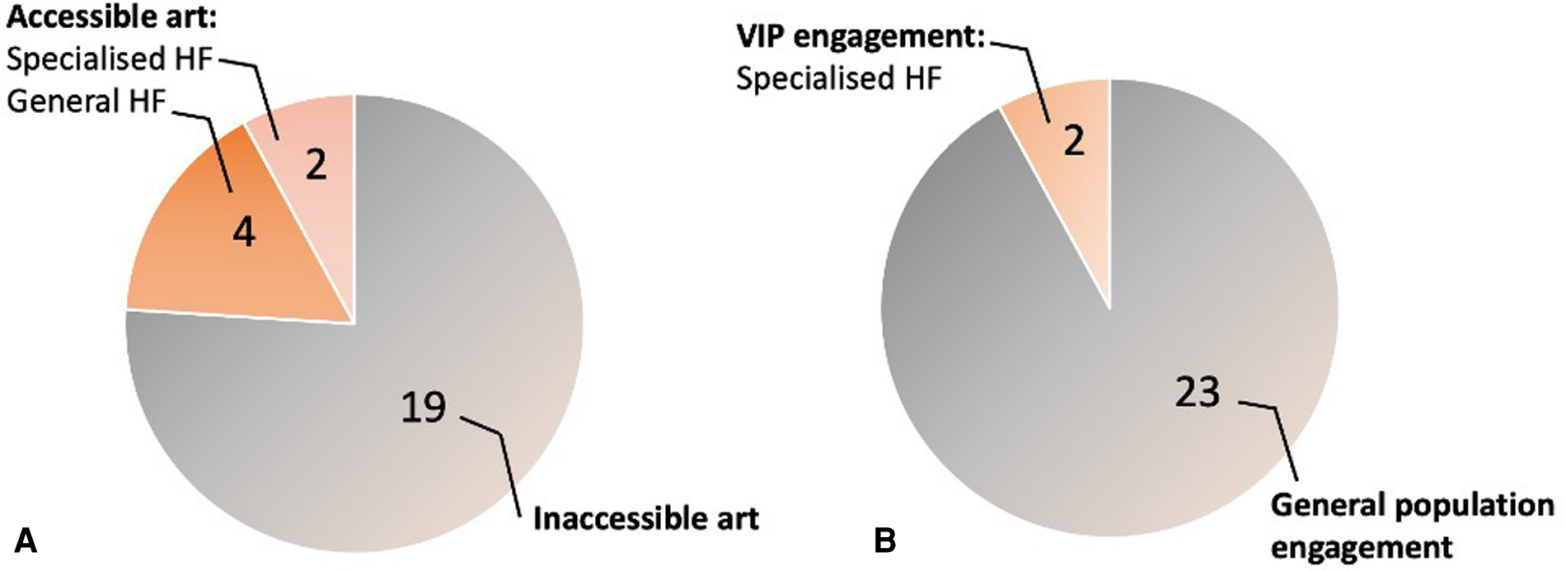
Findings on evaluation on accessibility for VIP of art programmes in HF. (**A**) Prevalence of the accessible art for VIP in healthcare facilities (HF). (**B**) Prevalence of user engagement in the commissioning of healthcare art projects.

The results of statistical analysis of accessibility of healthcare art programmes are the following (Figure 3). In terms of accessibility of arts, six healthcare facilities out of 25 included tactile or auditory modalities to at least one of their art pieces. Interestingly, two out of five accessible art projects were in specialised healthcare facilities that provided medical aid for sensory disabled individuals. Other four healthcare facilities were general hospitals which did not provide details if the art was made with intention of accessibility for VIP, although at least one of their art pieces had multisensory features. For example, one mental health facility provided tactile panels with intention to use it as an object for physiotherapy. Another interactive art piece with tactile features was created to improve communication between physicians and patients with hand injuries. In terms of VIP engagement, only two facilities, specialised hospitals mentioned above, collaborated with VIP. Nevertheless, all assessed healthcare facilities underlined that they invited patients and community members for either consultation, interviews or co-creation.

### Review of guidelines on art in healthcare facilities

A comprehensive review of 31 HBN guidance sources, along with supplementary documents for the main documents, as well as 4 supplementary documents focused on arts in healthcare settings, will be presented in two parts. The first part will cover the general requirements for arts in healthcare settings, while the second part will focus on the prevalence of the need for accessibility for VIP.

### General requirements for arts in healthcare settings

The majority of the HBN for various types of facilities comprised background material on the value of the arts and some commissioning and maintenance considerations, including securing funds, deciding on potential location and installation, and infection control. Some adaptations were necessary for the specifics of a particular clinical department and its users' needs. For instance, surgical and cancer treatment institutions received more focus on infection control, while mental health facilities received more attention for safety and art theme appropriateness. Interestingly, the most extensive information on art was provided in HBN for facilities for people with dementia as a part of the Dementia Friendly Environments Report. The guidance revealed which types, themes, locations, and scales of healthcare art can complement dementia-friendly design principles and improve the lives of patients with dementia.

Four additional documents provided information on various points needed in an art program. For instance, “*A prospectus for art and health”* was mainly focused on benefits that arts could bring and had project descriptions primarily held in the community rather than clinical settings. However, several case studies in healthcare facilities gave valuable information on how to launch a project, advice on the most effective ways of collaboration and recommendations on making art an integral part of the physical environment with attention to users' needs. Two other sources, “*The art of good health—using visual arts in healthcare”* and “*The art of good health—a practical handbook”*, provided information on participatory and receptive arts in healthcare settings. They complemented each other as one book was mainly structured as a step-by-step guide with different stages of art commissioning and maintenance, and another book included numerous case studies of art programmes across the UK. The final source, entitled “*Arts and community engagement in LIFT*,” contained interviews with prominent healthcare arts experts, providing valuable insights and case studies.

Based on the reviewed documents, the main points were summarised in three dimensions that could influence the final art project result: people, space, and artwork features (Table 1).

**Table 1:** Major dimensions and criteria identified in the review of healthcare arts guidelines.

Dimension	Criteria
People	Users of area/facility/department (patient groups, visitors, staff) Cultural diversity Local area Accessibility of theme (easy to relate)
Space	Purpose of space (public space, treatment/diagnostic room, private room) Location of artwork for good presentation and not creating any obstructions Cleaning and maintenance with particular focus on infection control Disabled access Sustainability Security
Artwork	Idea, concept (landscape, figural, portrait, abstract, etc.) Art form (photograph, painting, sculpture, etc.) Materials (paint, textile, wood, plastic, etc.) Quality of artwork

### Need for accessibility for VIP in healthcare facilities guidance

The source documents had only superficial information on the accessibility of arts. HBN did not include any references or guidelines regarding the provision of accessible arts or measures to enhance physical accessibility to arts. However, every HBN guidance document addressed the necessity of providing equal access for people of different abilities in sections on other design elements to adhere to the Equality Act 2010. In the four supplementary documents, only one source pointed out that there should be disabled access to artworks but without further details on how to provide that.

The present study reveals that the Health Building Note (HBN) contained scanty information on addressing the needs of users concerning arts. Specifically, the HBN's coverage on this matter was confined to two guidelines which focused on mental health facilities for children and adults. The main objective of these guidelines was to select art themes that did not pose any threat to the physical safety of the patients and did not have any adverse effect on their mental well-being. The four supplementary documents strongly emphasised the need to involve users in their projects. The proposed opportunities for collaboration included conducting discussions and workshops with both patients and staff to identify a specific art direction for the department. However, there were no details on the need to include VIP in collaborations and consultations.

### Identification of opportunities for healthcare art programmes

The healthcare facilities that were identified in Step 1 of the study as having accessible arts were invited to participate in interview surveys. A total of six facilities were contacted and invited to participate. Of the six facilities, three professionals replied to the invitations and agreed to take part in the online interview. The selected Trusts' projects are briefly introduced as case studies followed by the results of the interview surveys.

### Case studies of accessible healthcare art projects

The study included participation from three London trusts, each with art projects that offered accessible solutions. These included a multisensory room located in Guy's Hospital Cancer Centre from Guy's and St. Thomas Trust (GT), the Arts and Heritage programme in the Grafton Way Building offered through the University College London Hospital (UCLH), and interactive arts featured at Great Ormond Street Hospital (GOSH).

### Guy's hospital's cancer centre—the living room

The Living Room is a space for patients and visitors of Guy's Hospital Cancer Centre located on the ground floor of the building. The room provides an environment where patients and their families can escape the clinical walls and enjoy natural sounds from different parts of the world. The central area of the room provides some space for communication. The seats create separate listening zones with a personal screen to select a preferred soundtrack that can be listened to without interference with neighbouring zones. The listening zones create a multisensory experience for users, embedding ambient sounds with cosy tactile feelings of furniture fabric and wooden details. The furniture's suitability for the strict clinical requirements was carefully considered. The use of bamboo wood and textiles that are impervious to contaminants was found to strike a balance between meeting The Living Room's objectives and the Cancer Centre's infection safety requirements. Furthermore, it can be noted that the furniture's design is not traditionally institutional in appearance, which provided further aesthetic experience for its users.

### The UCLH arts and heritage programme—the Grafton way building

The UCL cancer and surgery hospital for advanced cancer therapies was created with patients in mind and provided a variety of art integrated into its design. The facility locates over a hundred artworks that bring patients a positive distraction from clinical routines. On the top of the roof is a terrace with fragrant herbs planted in the garden and pleasantly tactile stone sculptures of animals. Apart from that, the UCLH art programme provides art exhibitions with a series of on-site and online events, which helps to get a holistic experience by enjoying arts through guides, descriptions, and communication. Interestingly, all art collections are published on the UCLH website with details on each project. An accessible website policy enables people with sight problems to get all information on UCLH arts and events and know how to get them when in the hospital.

### Great Ormond street hospital—sight and sound centre

GOSH Sight and Sound Centre has an awarding art programme that was created to support children with sight and hearing impairments. World-renowned artists worked together with the design team, children and their parents to create sensory art pieces across the building. One of the most notable arts are an installation Pythagoras Stairs that creates vibrations and organ sound when climbing the stairs; a light-up Doll's House in the reception area that is an interactive wayfinding solution for children; and Past Lives and Future Tools—ceramic tiles in waiting and terrace areas that were designed together with children. The hygiene aspects of GOSH interactive arts were thoroughly considered in collaboration with the infection control team, and the appropriate materials for frequent cleaning were used for all artworks. The issue of sustainability was also followed across the GOSH arts program.

### Interview survey data from art professionals

**Figure 4:**
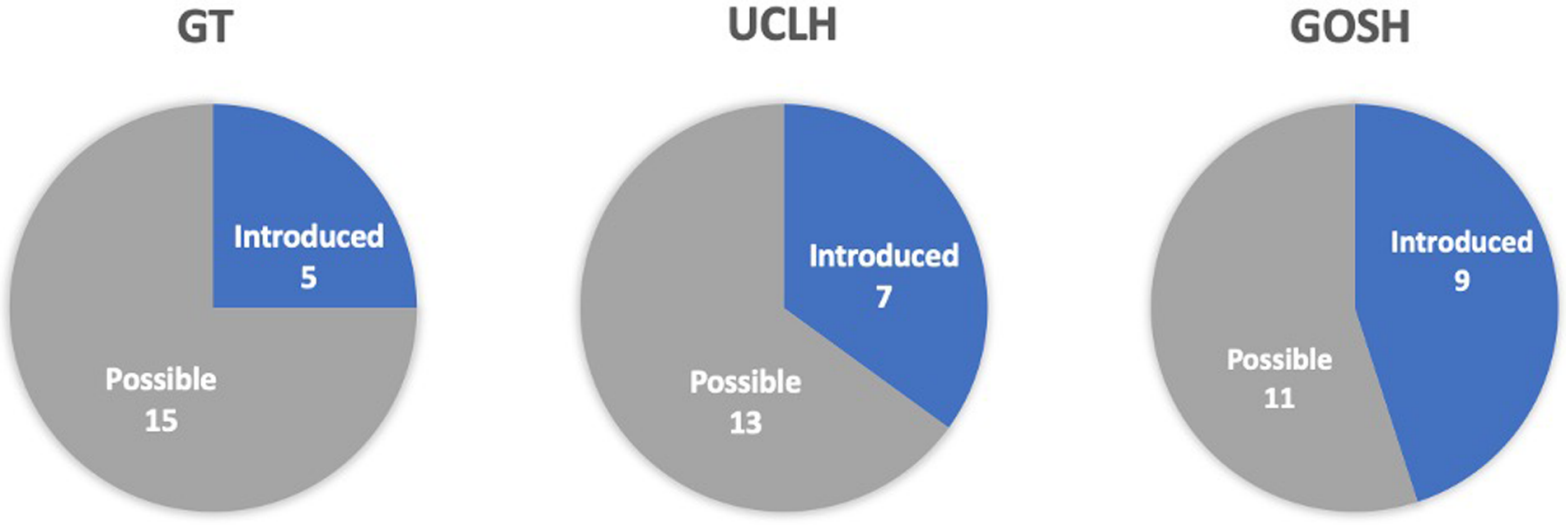
Summary of existing and possible opportunities for art programmes in three NHS trusts: GT, Guy's and St. Thomas; UCLH, University College London Hospital; GOSH, Great Ormond Street Hospital.

It is noteworthy that none of the 20 proposed activities were marked as impossible or uncertain (Figure 4). The overall summary of the identified activities is as follows: the GT art programme had 5 existing activities, with 15 identified as possible; in the UCLH, 7 activities were introduced and 13 were possible; and in the GOSH, 9 activities were introduced, with 11 identified as possible.

In more detail (Table 2), only the GOSH trust implemented one out of four activities outlined in Step 1, “planning for inclusion”, which involved consultations with VIPs. The other accessibility-related activities were categorized under Step 2, “information access”, where the GT and GOSH trusts implemented one activity each, while the UCLH implemented two. The GT had an accessible information policy, the GOSH trust had information available in accessible formats, and the UCLH promoted art services in accessible formats and created an accessible website. In Step 3, “improving access”, each trust's art programme had at least two accessible solutions. The GT trust had guided tours and a multisensory art project, while the UCLH trust had all solutions in the form of accessible art services, including guided tours, online access to collections, and audio guides. GOSH had the widest variety of accessible art services and art forms. None of the trusts had touch tours and handling sessions, and two out of three trusts, GT and GOSH, had multisensory projects. In Step 4, “welcoming visitors”, UCLH did not have any of the opportunities but marked them as possible. None of the trusts had training for staff on visual awareness in arts programmes. However, GT and GOSH trusts had accessible facilities and wayfinding solutions for VIP.

**Table 2:** Interview survey results of introduced and possible accessible opportunities for art programmes in three NHS trusts: GT, Guy's and St. Thomas; UCLH, University College London Hospital; GOSH, Great Ormond Street Hospital.

Step	Activity	Introduced			Possible		
		GT	UCLH	GOSH	GT	UCLH	GOSH
1. Planning for inclusion	Access policy	–	–	–	✓	✓	✓
	Access audits	–	–	–	✓	✓	✓
	Consultation with VIP	–	–	✓	✓	✓	–
	Evaluation	–	–	–	✓	✓	✓
2. Improving access: information	Accessible information policy	✓	–	–	–	✓	✓
	Promoting services	–	✓	–	✓	–	✓
	At the venue	–	✓	–	✓	–	✓
	Access guide: (1) visitor information, (2) inform on your collections, exhibitions or building	–	✓	–	✓	–	✓
	Information in a range of accessible formats	–	–	✓	✓	✓	–
	Accessible website	–	✓	–	✓	–	✓
3. Improving access: accessible art servises	Guided tours that describe collections	✓	✓	✓	–	–	–
	Events when a site, objects or works are described	–	–	✓	✓	✓	–
	Access to collections online	–	✓	✓	✓	–	–
	Audio-guides	–	✓	–	✓	–	✓
Improving access: accessible art forms	Touch tours or handling sessions	–	–	–	✓	✓	✓
	Representations of objects or images in tactile formats	–	–	✓	✓	✓	–
	Multi-sensory exhibits	✓	–	✓	–	✓	–
4. Welcoming visitors with sight problems	Training for all staff	–	–	–	✓	✓	✓
	Accessible physical environment	✓	–	✓	–	✓	–
	Information on physical layout	✓	–	✓	–	✓	–

## Discussion

### Accessibility of art programmes in British healthcare facilities

NHS facilities' objectives to comply with the Equality Act 2010 posed the need to adapt healthcare environments for individuals of different abilities. Given that art is an integral part of the healing environment, and that visual impairment has become a more widespread issue, it was needed to discover whether healthcare art is accessible for VIP. The research on British healthcare art programmes revealed that healthcare art programmes were predominantly inaccessible for individuals with sight problems. To explore ways to improve the current state of arts accessibility, it was suggested that a crucial step would be to engage in collaborative efforts with stakeholders, including VIP (66). The study found VIP involvement in art commissioning process in only two medical facilities dedicated to eye-related conditions, which had both accessible art and VIP engagement. The other four programmes with accessible art did not prioritize VIP accessibility explicitly, such as a programme for multisensory art for children with mental health conditions or one for individuals with hand injuries. Hence, while involving VIP can enhance the arts accessibility, it should be noted that focusing on other health conditions can also contribute to the goal of making art more accessible for VIP. This supports the fact that integrating multiple sensory experiences in art can benefit not only VIPs but also the general population by providing a more immersive experience (43).

### Healthcare arts regulations influencing accessibility of healthcare arts

The document review of HBN and four supplementary documents on healthcare art provided numerous guidance on healthcare arts that were divided into three dimensions: people, space, and artwork. The first identified dimension in the healthcare guidance review was focus on people. The review has shown that art programmes would be more successful and beneficial for their users if they consider a diverse population's needs, tastes, and cultural views. As we have seen from the above analysis, artworks are still not adapted for VIP needs, which raises the question of why this strong message to be user-oriented does not help make arts more accessible. The answer might be not only on the healthcare organisation level but also on the societal level. Visual impairment remains an undercovered topic in society, and VIP feel discriminated against in normal life activities (32, 34). Thus, visual impairment awareness could help members of society become familiar and foster understanding and respect for VIP in their communities (82). This could subsequently influence how hiring process of user groups in terms of their diversity is happening. Therefore, involving the general population in discussions of artwork for VIP could help the shift the salience of VIP's experiences.

The second dimension extracted from healthcare art requirements were considerations of the space or physical healthcare environment. Good practices emphasised the need of early-stage considerations regarding such issues as adequate presentation of an artwork, enough space to allow staff and patients movements, infection control and sustainability. In the context of incorporating accessible artworks, such as tactile and multisensory art pieces, into healthcare facilities requires early-stage consideration of physical space, installation location, and artwork presentation, while also taking into account patient and staff flows to prevent obstruction. In addition, infection safety must also be considered when integrating tactile and multisensory art pieces within healthcare spaces, as highlighted in recent literature review (50, 51). Therefore, collaboration between design and infection control teams is essential to ensure the safety and efficacy of accessible artworks in healthcare settings. Thus, early-stage consideration of the arts and the requirements of space for them, would stimulate the accessibility of arts as there would be more chances to find solutions on how to put accessible arts in clinical spaces with strict safety restrictions.

The third dimension outlined requirements for the features of artwork, including its theme, materials, and quality, to maximize its therapeutic effects in a healthcare environment. It emphasized the need for an art scheme to align with the objectives and functions of the space and the needs of users. This suggests that the selection or creation processes of art for healthcare facilities should consider the preferences of user groups as well as the unique characteristics of the clinical space, both of which are influenced by the abovementioned factors that require investigation. Moreover, it remains unclear which features of accessible art are applicable in healthcare facilities due to the lack of specific recommendations for VIP. Although adaptations of accessible art, such as tactile art and audio solutions, have been used in museums and galleries, their efficacy in healthcare settings with VIP cohorts is unknown. While art provides aesthetic appreciation regardless of its location, healthcare spaces' unique characteristics and the special needs of users, who may experience stress due to their physical condition, may require different themes, materials, and other art features. Patients' needs differ from those of visitors to galleries and museums. Therefore, research is necessary to determine which features of accessible art, are best suited for the built environment of healthcare facilities in terms of their therapeutic effects for VIP, as well as safety measures for physical environment.

### The question of accessibility in healthcare design guidelines

The Health Building Notes recommend adhering to the Equality Act 2010 for different built environment elements and provide general guidance on providing disabled access to artworks. However, specific instructions on achieving accessibility of arts are lacking. Although co-creation in arts commissioning is emphasized, there is no mention of involving people with disabilities, including VIP, in user groups. Given that VIP are more likely to require accessibility adaptations in different medical facilities due to their multiple chronic conditions (31, 33), it is necessary to explore why they are excluded from co-creation in healthcare art programmes. The findings suggest that the current guidance for healthcare art schemes is insufficient in improving accessibility, which is consistent with the results of the analysis of healthcare art schemes in Step 1. Therefore, a revision of the guidance that incorporates evidence-based design and art expertise is necessary to provide clear instructions for artists, art managers, and other professionals working on healthcare art.

### Identified opportunities to increase accessibility of arts

One of the noteworthy discoveries was that all available artistic activities presented to healthcare art experts were considered possible, provided they had not yet been implemented in the art programmes of the respective healthcare trusts. This finding provides a basis for initiating a discourse on why the activities listed in the checklist have yet to be integrated into healthcare institutions and exploring potential measures to introduce them. Moreover, the solutions that already exist in selected trusts are discussed as potential for other healthcare facilities.

Another important finding was that none of the trusts had key “**planning for inclusion**” activities that could stimulate effective accessible solutions, except for GOSH that had consultations with VIP. The access policy supported by access audits, consultations with VIP and evaluation would help to create a clear action plan understandable for all team members (53). Indeed, developing access policy and plan would further help to follow the Equality Act 2010 and, therefore, healthcare facilities' objectives of inclusivity of care. Interestingly, the GOSH trust that had consultations with VIP had the highest number of accessible arts and related activities. Thus, introducing an access policy with a clear plan and supportive activities like consultations with VIP, audits and evaluations could increase accessibility of healthcare art programmes.

**“Improving access to information”** was the next set of opportunities for healthcare art programmes to increase accessibility. While each evaluated trust had some solutions to increasing information access, the UCLH trust had the highest rate of them, including services promotion and an accessible website that helped VIP reach all artworks located in hospitals, prepare for the hospital visit, and attend on-site and online events. These implementations align with the Accessible Information Standard for all trusts that many NHS hospitals still fail to follow (10). Having the example of art programmes with accessible information, such as UCLH, other trusts could adopt that experience. It can be argued that the solutions being examined appear to present fewer challenges to healthcare environments, as they do not necessitate any physical modifications to the existing space to introduce accessible art forms. Hence, it can be concluded that the development of an accessible information policy, the promotion of art services, and the provision of information in diverse accessible formats, such as accessible websites, appear to be viable solutions that can be adopted by any healthcare trusts seeking to enhance the accessibility of their art programmes.

Another interesting finding was that not only arts by themselves could be adapted to provide access, but also some **art services** like guided tours, on-site and online events, audio guides, and access to collections online. Some of these solutions were already introduced in evaluated trusts, with guided tours in each. Considering the challenges associated with the underexplored aspects of accessible art forms in healthcare settings, art description events and online services emerge as a feasible opportunity for the already established art programmes across NHS Trusts. Working in collaboration with relevant charitable organisations such as Vocaleyes would ensure the quality of services and might help address funding issues (53, 54, 70, 71).

With respect to the proposed **accessible art forms**, none of the hospitals under study offered touch tours, although GOSH had incorporated representations of art in tactile formats. Several factors could potentially account for this disparity. Firstly, GOSH had engaged in consultations with VIP, whereas the lack of tactile arts in the other two trusts may be attributed to their lack of collaboration with VIP during the art commissioning process. Secondly, strict infection control regulations might have constrained art professionals from introducing any arts that could potentially compromise the infection control protocols in place. Nonetheless, potential solutions for introducing tactile arts while adhering to COVID-19 safety restrictions identified in the literature review (50, 51, 53) could be taken into consideration for implementation in healthcare facilities. Moreover, the availability of multisensory exhibits in both GOSH and GT trusts presents an opportunity for the integration of accessible forms of arts in other healthcare settings. To address concerns regarding direct contact infection transmission, materials such as bamboo and impermeable textiles that reduce surface infection transmission have been found to be effective. However, further research is needed to establish the safety of such materials. Notably, GOSH had devised an alternative solution through the sound and vibration art installation *Pythagoras Stairs* which may alleviate concerns about infection safety. Consequently, despite the inherent challenges of operating in a healthcare environment, there is ample scope for creative implementation of multisensory art forms in healthcare settings.

“**Welcoming visitors**” step shows sheds light on additional factors that could enhance the accessibility of arts in healthcare settings. The provision of staff training, improvements in the built environment, and the implementation of accessible wayfinding systems are some of the considerations that could potentially enhance the accessibility of healthcare art programmes. The interviews revealed two out of three trusts had accessible physical environments and wayfinding solutions for VIP. However, none of the trusts had provided visual awareness training to their staff, and UCLH did not have any of the activities. It is noteworthy that the absence of such features in UCLH does not necessarily imply that the facility lacks the requisite accessible solutions, given the general requirement for healthcare facilities to adhere to the provisions of the Equality Act 2010. The lack of collaboration among professionals involved in healthcare facility design may account for the limited progress towards achieving the objective of providing inclusive and accessible design and services. Providing visual awareness training for all staff members could promote collaboration and encourage changes in neighbouring sectors of healthcare design. It is worth emphasizing that healthcare arts should be an integral part of hospital design and facility objectives. Hence, accessibility to arts should not be viewed in isolation but rather as part of a comprehensive design scheme and supporting services. Establishing a clear objective of accessibility for VIP in healthcare facilities can significantly contribute to enhancing the accessibility of arts.

## Conclusion

NHS healthcare settings have been required to adapt to the needs of people with disabilities under the Equality Act 2010. Considering the significance of arts in the therapeutic environment and the growing population of visually impaired individuals, it was crucial to assess the accessibility of healthcare art and explore strategies to enhance it. The study drew on existing knowledge of accessible arts in museums and galleries for the blind and partially sighted to evaluate healthcare arts and the efficiency of healthcare facilities guidelines. Additionally, interviews with art professionals from NHS trusts with accessible art programmes were conducted to identify opportunities for increasing accessibility.

The research has demonstrated that healthcare art programmes in the UK are predominantly inaccessible to visually impaired individuals and lack adequate procedures to improve it. Most healthcare arts did not have multisensory alterations and did not include visually impaired individuals in arts commissioning process. The evaluation of guidelines for healthcare facilities revealed a lack of attention to the requirements of visually impaired individuals in the overall guidance provided for art and the process of commissioning art, as well as in the physical space itself. The insufficient recommendations in healthcare facility guidelines require their revision with evidence-based alterations.

The findings of this research have identified various practical applications that can assist healthcare providers and inform policymakers in developing guidelines for healthcare art programmes. Firstly, improving arts accessibility for individuals with visual impairments can be achieved through involving VIP and general population in discussions of accessible artworks, as well as considering art projects at early stages alongside space requirements. For the arts, audio art services and online access to artworks could be feasible for many trusts without the need for physical space alterations. Secondly, implementing an access policy and accessible information policy, including the provision of information in diverse accessible formats, would promote equality and compliance with the Equality Act 2010 and the Accessible Information Standard in NHS facilities. Collaboration among professionals working on healthcare design and providing visual awareness training for all staff members is essential to improve accessibility to arts and promote changes in neighbouring sectors of healthcare design. Lastly, arts accessibility should be integrated into the general design scheme and services of healthcare facilities, and a clear aim of accessibility for VIP should be established to enhance the accessibility of healthcare arts.

The exploratory research conducted in this study has several limitations and opportunities for future research. One of the main limitations is that the analysis of healthcare art programmes was based on secondary data, which may not accurately represent the current state of accessibility of art in healthcare facilities. To address this, future studies could collect primary data through surveys, interviews, and observations, allowing for a more comprehensive assessment. Additionally, further research is needed to investigate the common needs of users and the impact of accessible art on diverse population groups beyond those with visual impairments, as multisensory art may have positive effects on a larger population. Understanding how different demographic groups engage with art will provide insights for designing more inclusive art programmes. Finally, there is a need to extend case studies beyond large London trusts to include smaller trusts across the UK and diversify healthcare contexts. Conducting this step before exploring multisensory art features for healthcare facilities will ensure that recommendations for different trust contexts are informed by a broader scope of case study insights. Finally, the research was conducted within a single cultural framework, the United Kingdom, thereby suggesting the potential for broader application across different nations.

## Data Availability

The original contributions presented in the study are included in the article/Supplementary Material, further inquiries can be directed to the corresponding author.
