# Supplementary material for: Accessible art in healthcare facilities: exploring perspectives of healthcare art for visually impaired people
Source: Front Med Technol. 2023 Oct 23;5:1205361. doi: 10.3389/fmedt.2023.1205361 (PMC10627155; doi:10.3389/fmedt.2023.1205361)
Supplement: Supplementary file 1 [file Table1.docx]

| **Survey 'Accessible Art for VIP'** | | | **[Trust Name]** | | | |
| --- | --- | --- | --- | --- | --- | --- |
|  |  |  | **Introduced** | **Not introduced** | | |
| **Stage** | **Name** | **Activity** |  | **Possible** | **Impossible** | **Difficult**  **to say** |
| **1** | **Planninig for inclusion** | develop access policy |  |  |  |  |
|  |  | access audits - to identify all the barriers and hazards |  |  |  |  |
|  |  | consultation with VIP |  |  |  |  |
|  |  | evaluation |  |  |  |  |
| **2** | **Improving access: accessible art servises** | guided tours that describe collections |  |  |  |  |
|  |  | special events when a site, objects or works are described |  |  |  |  |
|  |  | providing access to collections online |  |  |  |  |
|  |  | audio-guides |  |  |  |  |
|  | **Improving access: accessible art forms** | touch tours or handling sessions |  |  |  |  |
|  |  | representations of objects or images in tactile formats |  |  |  |  |
|  |  | multi-sensory exhibits |  |  |  |  |
| **3** | **Improving access: information** | adoption accessible information policy |  |  |  |  |
|  |  | promoting services |  |  |  |  |
|  |  | information in a range of accessible formats |  |  |  |  |
|  |  | accessible website |  |  |  |  |
| **4** | **Welcoming visitors with sight problems** | training for all staff |  |  |  |  |
|  |  | physical environment |  |  |  |  |
|  |  | information of physical layout |  |  |  |  |
